# Overtreatment of cruciate ligament injuries

**DOI:** 10.3109/17453674.2011.555374

**Published:** 2011-02-10

**Authors:** Sverre Løken, Asbjørn Årøen, Lars Engebretsen

**Affiliations:** Orthopaedic Department, Oslo University Hospital, Oslo, Norway

*Sir*–In an editorial in the October issue of Acta Orthopaedica 2010, Per Aspenberg (Aspenberg 2010) comments on the article recently published in New England Journal of Medicine (Frobell et al. 2010). We congratulate the authors on this well performed randomized controlled trial on the treatment of ACL injuries were they found that rehabilitation + ACL reconstruction was not superior to rehabilitation + optional delayed ACL reconstruction when using the mean of 4 of the 5 subscales of the KOOS score at two years as the primary outcome variable. Aspenberg concludes in his editorial that most patients who are operated on early after ACL injury undergo the procedure in vain. However, in our opinion the difference in meniscal surgery between the groups in Frobell's study may indicate the opposite. Preservation of the menisci is a key factor in preventing later osteoarthritis in ACL-deficient knees as previously shown in another study, also from Lund University, Sweden (Neumann et al. 2008). As shown in table D in supplementary material (Frobell et al. 2010) the total number of treated menisci at baseline was 34 in the early ACL reconstruction group versus 21 in the rehabilitation + optional delayed ACL reconstruction group. During follow up these numbers were 6 versus 29 respectively (<0.001). The authors say that meniscal tears were managed more aggressively in the subjects assigned to early ACL reconstruction and were more likely to be left untreated in the subjects assigned to rehabilitation plus optional delayed ACL reconstruction, and believe that this difference probably explains the greater frequency of meniscal surgery during follow-up in the latter group. In our view another possible explanation could be that meniscal tears that were small and non-symptomatic in the early phase became larger and symptomatic with time in the non-reconstructed knees. Thus, an injury that could be treated with either repair, or a small resection or left untreated at the time of an early ACL-reconstruction could end up as a large bucket handle tear leading to a subtotal menisectomy in an unstable knee. Secondly, the risk of developing new injuries to the menisci and articular cartilage may be higher in a non-reconstructed knee. This is supported by registry data (Granan et al. 2009). Finally, the difference in the frequency of meniscal injuries between the groups may increase further with longer follow-up time. In summary, Frobell's study could just as well support early reconstruction to protect the menisci. We look forward to the long term follow-up in this study. If the frequency of meniscal tears continue to accumulate at the same rate in the non-reconstructed knees this study may end up showing that most early ACL reconstructions are not performed in vain.
