# Supplementary material for: Overtreatment of cruciate ligament injuries
Source: Acta Orthop. 2011 Feb 10;82(1):122–3. doi: 10.3109/17453674.2011.555374 (PMC3230010; doi:10.3109/17453674.2011.555374)
Supplement: Related texts

# Letter: Overtreatment of cruciate ligament injuries

**Published:** 2011-02-10

**Authors:** Per Aspenberg

**Affiliations:** Department of Orthopaedics, IKE, Faculty of Health Sciences, Linköping University, Swedenper.aspenberg@liu.se

*Sir*–The main goal of cruciate ligament reconstruction in the sports medicine community has been to enable the patients to go back to a high functional level (often sports activities) in a short time. The study by Frobell et al. shows that, for this end, surgery is need-less for most patients if appropriate physiotherapy is provided. In that respect, I maintain that most patients may be operated on in vain, and that beginning with a structured training program is the treatment of choice for a vast majority.

The letter from Løken et al. now brings up the subject of long-term results, i.e. the risk of osteoarthritis. This is a commendable and much awaited change of focus in the cruciate ligament replacement field. It remains to see whether reconstruction can prevent – or possibly cause – osteoarthritis. Acta Orthopaedica has asked Drs Lohmander and Frobell to answer the letter regarding this point.

# Letter: Overtreatment of cruciate ligament injuries

**Published:** 2011-02-10

**Authors:** L Stefan Lohmander, Richard B Frobell

**Affiliations:** Department of Orthopedics, Clinical Sciences Lund, Lund University, Sweden, and Research Unit for Musculoskeletal Function and Physiotherapy and Department Orthopaedics and Traumatology, University of Southern Denmark; Department of Orthopedics, Clinical Sciences Lund, Lund University, Sweden

*Sir*–We thank Dr. Løken and colleagues for their interest in our study on anterior cruciate ligament (ACL) injuries. At 2 years of follow-up our randomized controlled study showed no advantage of early ACL reconstructive surgery with structured rehabilitation over that of structured rehabilitation only with optional reconstruction ‘as needed’, as monitored by KOOS (Frobell et al. 2010). Moreover, we found no difference in any of the secondary outcomes, or in the number of meniscal surgeries during the 2-year follow-up. We share the concern of Løken and colleagues for the long-term outcome after injury to the anterior cruciate ligament (ACL), in particular the development of osteoarthritis, and continue to monitor this patient group long term by patient-reported outcomes, activity level, plain radiographs, magnetic resonance imaging, and biomarkers. Results for these outcomes will be reported.

Løken and colleagues raise three points. Firstly, they suggest that the literature shows that a functionally intact meniscus may be important to prevent future osteoarthritis in the ACL-injured knee. We agree on the likely importance of a functionally intact meniscus to prevent the development of knee osteoarthritis, but note that not all reports on the ACL-injured knee are consistent with this hypothesis.

Secondly, they suggest that the risk of re-injury may be increased in the non-reconstructed knee. This is certainly possible, but all studies published so far with the exception of one (Frobell et al. 2010), are confounded by indication. We agree that the registry study of Granan (Granan et al. 2009) shows that the frequency of observed cartilage and meniscal damage increases with time after injury. This is to be expected, has been reported (Roos et al. 1995, Lohmander et al. 2007), and may be interpreted as early-stage osteoarthritis development. In the Granan study, no results were provided to report later cartilage and meniscus status by follow-up of those already reconstructed, so we are unable to draw any conclusions on the benefit of reconstruction on these outcomes from their study. Further, no data are available for those not included in the registry, and meniscus injury caused by the initial trauma is not reported. Recent observational studies find no difference in later osteoarthritis between those reconstructed or not (Lohmander et al. 2007, Meuffels et al. 2009).

Thirdly, Løken and colleagues suggest that a difference in meniscal surgery rate between our study groups may develop with time. This is certainly possible. We found no difference between the two study groups in the number of meniscal surgeries at two years. Whether differences will show at later times remain to be reported. Until then we can only speculate.

A high proportion of those with an ACL tear become ‘young patients with old knees’. The rate of osteoarthritis development after these injuries remains a major clinical challenge: with regard to preventing these injuries, preventing osteoarthritis developing after the injury, and treating severe osteoarthritis in the young and active patient. We encourage further basic research on disease mechanisms, randomized trials to identify the best treatments, and large and long-term registry studies with minimal loss to follow-up to monitor long-term outcome and complications. In these studies, patient reported outcomes are central, other outcomes such as radiographic changes or numbers of surgeries are at best surrogates.

## References

[CIT0001] Aspenberg P (2010). Overtreatment of cruciate ligament injuries. Acta Orthop.

[CIT0002] Frobell RB, Roos EM, Roos HP, Ranstam J, Lohmander LS (2010). A randomized trial of treatment for acute anterior cruciate ligament tears. N Engl J Med.

[CIT0003] Frobell RB, Roos EM, Roos HP, Ranstam J, Lohmander LS http://www.nejm.org/doi/suppl/10.1056/NEJMoa0907797/suppl_file/nejmoa0907797_appendix.pdf.

[CIT0004] Granan LP, Bahr R, Lie SA, Engebretsen L (2009). Timing of anterior cruciate ligament reconstructive surgery and risk of cartilage lesions and meniscal tears: a cohort study based on the Norwegian National Knee Ligament Registry. Am J Sports Med.

[CIT0005] Lohmander LS, Englund M, Dahl LL, Roos EM (2007). The long-term consequence of anterior cruciate ligament and meniscus injuries: Osteoarthritis. Am J Sports Med.

[CIT0006] Meuffels DE, Favejee MM, Vissers MM, Heijboer MP, Reijman M, Verhaar JAN (2009). Ten year follow-up study comparing conservative versus operative treatment of anterior cruciate ligament ruptures. A matched-pair analysis of high level athletes. Br J Sports Med.

[CIT0007] Neuman P, Englund M, Kostogiannis I, Fridén T, Roos H, Dahlberg LE (2008). Prevalence of tibiofemoral osteoarthritis 15 years after nonoperative treatment of anterior cruciate ligament injury: a prospective cohort study. Am J Sports Med.

[CIT0008] Roos H, Adalberth T, Dahlberg L, Lohmander LS (1995). Osteoarthrosis of the knee after injury to the anterior cruciate ligament or meniscus - the influence of time and age. Osteoarthritis Cartilage.
